# Osteoporosis Screening Disparities among Ethnic and Racial Minorities: A Systematic Review

**DOI:** 10.1155/2023/1277319

**Published:** 2023-04-24

**Authors:** Anoush Calikyan, Jillian Silverberg, Katherine M. McLeod

**Affiliations:** ^1^Frank H. Netter MD School of Medicine at Quinnipiac University, North Haven, CT, USA; ^2^University of Connecticut Health Sciences Library, Farmington, CT, USA

## Abstract

**Background:**

Osteoporosis is a preventable disease that is simple and cost-effective to screen based on clinical practice guidelines, yet many patients go undiagnosed and untreated leading to increased burden of the disease. Specifically, racial and ethnic minorities have lower rates of dual energy absorptiometry (DXA) screening. Inadequate screening may lead to an increased risk of fracture, higher health care costs, and increased morbidity and mortality disproportionately experienced by racial-ethnic minority populations.

**Purpose:**

This systematic review assessed and summarized the racial and ethnic disparities that exist for osteoporosis screening by DXA.

**Methods:**

Using terms related to osteoporosis, racial and ethnic minorities, and DXA, an electronic search of databases was performed in SCOPUS, CINAHL, and PubMed. Articles were screened using predefined inclusion and exclusion criteria which dictated the final articles used in the review. Full text articles that were selected for inclusion underwent quality appraisal and data extraction. Once extracted, data from the articles were combined at an aggregate level.

**Results:**

The search identified 412 articles. After screening, a total of 16 studies were included in the final review. The overall quality of the studies included was high. Of the 16 articles reviewed, 14 identified significant disparities between racial minority and majority groups and determined that the eligible patients in racial minority groups were less likely to be referred to DXA screening.

**Conclusion:**

There is a significant disparity in osteoporosis screening among racial and ethnic minorities. Future efforts should focus on addressing these inconsistencies in screening and removing bias from the healthcare system. Additional research is required to determine the consequence of this discrepancy in screening and methods of equitizing osteoporosis care.

## 1. Introduction

Osteoporosis is a bone disease characterized by decreased bone density and mass, deterioration of bone tissue, and disrupted microarchitecture of bone [1, 2]. Osteoporosis poses both a major economic burden and a major public health concern worldwide [3, 4]. In the United States, approximately 54 million adults aged 50 and older have osteoporosis or are at risk due to low bone mass, and it is estimated that 1 in 2 women and nearly 1 in 4 men aged 50 and older will experience a fragility fracture in their remaining lifetime [3]. Similarly, the prevalence of osteoporosis-related fractures globally is a concern, with 1 in 3 women and 1 in 5 men aged over 50 impacted in their lifetime, leading to a significant increase in morbidity and mortality, reduced quality of life, and burden of healthcare costs [5]. While osteoporosis has traditionally been viewed as a chronic condition primarily among White women; the research literature continues to point to the growing prevalence of osteoporosis among people of color globally [6]. Hispanic women have the highest increase in osteoporosis prevalence, while the disease is severely under-recognized in African American populations [6]. Despite the consequences of osteoporosis to all, there is a clear disparity in care among racial and ethnic minorities.

The recognition and care for patients with osteoporosis begin with primary prevention of the disease through screening for osteoporosis in asymptomatic individuals. There are well-established clinical practice guidelines for the prevention and management of osteoporosis, including dual energy absorptiometry (DXA) to determine bone mass density (BMD) for screening and clinical diagnosis of the disease [7]. The World Health Organization (WHO) recommends DXA screening for women 65 years of age and older or for women 50–64 years of age with clinical risk factors, men 70 years of age and older or men 50–69 years of age with clinical risk factors, or anyone who has broken a bone after age 50 [4]. Screening for osteoporosis is essential to ensure early intervention of fracture risk by lifestyle modifications and/or pharmacological treatment when necessary. However, despite clinical practice guidelines and the clear benefits of screening, there is a well-documented care gap in osteoporosis screening for men and women meeting criteria, and this care gap is particularly prevalent among ethnic and racial minorities. For example, Hamrick and colleagues found that 29.8% of African American women versus 38.4% of White women were referred for DXA screening by their primary care providers, of which only 20.8% versus 27% underwent screening, respectively [8]. The National Osteoporosis Risk Assessment (NORA), a study on osteoporosis among postmenopausal women, also identified a higher prevalence of undiagnosed African American postmenopausal women compared to their White counterparts [9]. Neuner and colleagues found that over a two-year period, non-Hispanic Black and Hispanic women were 48% and 34% less likely to receive bone density testing prior to fracture than non-Hispanic White women, respectively [10]. These suboptimal screening rates can lead to even greater morbidity and mortality as often the disease is not diagnosed until a fragility fracture has already occurred. For example, Hispanic men and women were found to have increased rates of fragility fracture due to undiagnosed osteoporosis, especially when compared to their White counterparts [11]. Studies also indicate lower rates of DXA screening among racial and ethnic minorities, even after fracture, leading to lower treatment rates [6, 11–14]. As a result, many individuals go untreated and experience a greater proportion of the burden from osteoporosis-related fractures, even though patients who have racial and ethnic nonminority status in their population have a higher prevalence of osteoporosis [6, 13].

A current review of the literature is needed to globally increase awareness and compliance to osteoporosis screening clinical practice guidelines by clinicians among patients of racial and ethnic minority status and to inform future research and recommendations. The purpose of this systematic review was to provide a summary of global racial and ethnic disparities in osteoporosis screening in an effort to increase awareness and highlight the need for improving the delivery of clinical care surrounding osteoporosis.

## 2. Methods

A systematic review was performed following PRISMA (Preferred Reporting Items for Systematic Review and Meta-Analysis) guidelines [15]. The protocol, delineating the search strategy, methods of quality assessment and analysis, and the inclusion and exclusion criteria were registered with PROSPERO (ID# CRD42020205587). Two reviewers (AC and KM) independently conducted the screening of literature for inclusion and exclusion criteria and the methodological quality assessment for included studies.

### 2.1. Search Strategy

An electronic search of relevant articles was carried out using the following databases: SCOPUS, CINAHL, and PubMed. In addition, a grey literature search was conducted to identify eligible ongoing and unpublished studies, such as dissertations. Reference lists of eligible articles were also searched to capture as many articles as possible for screening. To determine search terms, reviewers worked with a medical librarian to decide which combination would generate the most relevant documents for review. The following search terms were used to search all databases:

*(Osteoporosis OR postmenopausal osteoporosis OR age-related Osteoporosis) AND (screening OR detection OR prevention OR testing OR diagnosis OR identification OR DEXA screening OR bone mineral density screening OR bone densitometry testing) AND (ethnic minorities OR racial minorities OR people of color OR minority groups OR minority health OR health disparities OR prevention disparities OR Health Care Disparities)*

The search was restricted to articles published between January 1997 and October 2020. This start date was inclusive of when osteoporosis clinical practice guidelines were first implemented.

### 2.2. Selection Criteria

Eligibility criteria were defined according to the PICO framework. Population: men and women 50 years of age and older who meet clinical indications for osteoporosis screening and/or were referred for BMD screening by a healthcare provider and/or experienced a fragility fracture; intervention: racial and ethnic minority status; control: racial and ethnic nonminority status; outcome: referral for BMD screening by DXA and/or BMD screening by DXA. Study designs considered included observational studies, specifically cross-sectional, cohort, and case control studies that assessed BMD screening. Randomized control studies were considered; however, they did not answer the study question and were thus not included in the final review. Case reports and reviews were excluded. Only studies conducted in a clinical setting, using a medical database or population-based administrative health data, were included. Studies conducted in all countries and continents were eligible in the search. Studies assessing imaging modalities other than DXA were excluded. Studies that only assessed racial or ethnic differences in BMD (T-score) or fracture rates were also excluded.

To select the articles that were included in the review, two reviewers independently reviewed inclusion and exclusion criteria to screen the initial list of articles. Criteria can be found in Table 1. Two reviewers (AC and KM) first applied these criteria to 412 titles and abstracts to create a primary list of sources. This information was recorded in COVIDENCE, a web-based platform used to support efficient management of systematic reviews. Next, the reviewers applied inclusion criteria to 47 full text articles to create the final list of papers that were included in the review. At each step, reviewers were blinded to each other's decision until the screening was concluded. If reviewers agreed over the list of papers, the study would continue. Disagreement between reviewers over any papers was discussed and resolved, and there was no need for an additional reviewer. The screening decisions and any text or data that was used from the article to make the screening decision were recorded in COVIDENCE. The final review included 16 articles.

### 2.3. Data Extraction

Two reviewers independently extracted data from these included articles. Reviewers were again blinded to each other during data extraction. Discrepancies were identified and resolved through discussion, and a third party was not needed to come to a resolution. The extracted data were recorded in an Excel spreadsheet. Data collected included study characteristics (study objective, design, year study was conducted, sample size, location, and the exposure details, including the ethnic/racial status that was studied), population characteristics (age, sex, socioeconomic status, geographical residence, education, and any clinical risk factors identified for osteoporosis screening), and outcomes (referral for BMD screening by DXA and completed BMD screening by DXA).

### 2.4. Quality and Risk of Bias Assessment

Bias and quality assessment at each stage of the review was addressed independently by two independent reviewers (AC and KM). At each stage, reviewers first recorded their findings. Next, results were compared between reviewers to ensure consensus, and no third party was required to settle any discrepancies.

The quality of articles was assessed using the Newcastle-Ottawa Scale for Case Control and Cohort studies. Risk-of-bias assessment focused on cohort selection, outcome ascertainment, attrition, and adjustment for confounding variables. A modified Newcastle-Ottawa Scale was used to assess cross-sectional studies.

### 2.5. Analysis and Synthesis of the Literature

Selected articles included observational studies: specifically, cross-sectional, cohort, and case control studies. No experimental studies were included in the articles selected for further analysis. Selected observational studies were then read by the two reviewers (AC and KM). These data were summarized at an aggregate level.

## 3. Results

**Figure 1:**
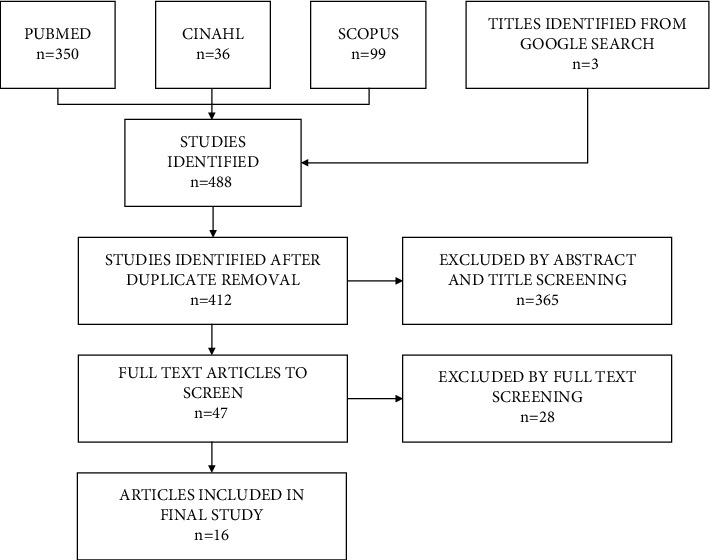
Flow chart of identification and selection of studies.

The initial search for articles identified 412 original articles. After screening abstracts and full text articles using the predefined inclusion and exclusion criteria, 16 articles were chosen for further analysis (Table 1). These studies went through quality appraisal and data extraction. The study selection process can be found in Figure 1.

### 3.1. Summary of Included Studies

Features of the included studies are summarized in Table 2. Of the 16 articles chosen, 10 articles were cohort studies [8, 10, 16–23], 5 were cross-sectional studies [24–28], and 1 study involved the use of a case control format [29]. Fourteen of the sixteen articles were based in the United States [8, 16–26, 29], while the other 2 were based in Israel [27, 28]. Racial minority groups involving Black patients were the most studied, where 13 studies examined the difference between DXA screening in between Black and White patients [8, 16, 19, 21–26, 29]. Four studies investigated ethnic minority groups including Hispanic patients [10, 17, 18, 20], one identified Arab patients as a racial-ethnic minority group [28], and one recognized Ethiopians as a racial-ethnic minority group [27].

### 3.2. Study Quality

Quality appraisal scores of chosen articles are summarized in Table 3. The quality of the selected studies generally was high, ranging from a score of 7 to 9, out of a possible highest score of 9.

### 3.3. Study Setting and Participants

Of the 16 articles, 14 studies were based in the US, while 2 were performed in Israel [27, 28]. Two articles included data from the United States Preventative Service Task Force (USPSTF) electronic health records [17, 20], 1 article from Medicare electronic health records [19], and 13 from various health centers in the United States and Israel [8, 10, 16, 18, 21–29]. Most studies focused on women aged 65 and older, though 4 of those studies included men [18, 19, 21, 22] with only 1 study focusing solely on men over 65 years of age [22]. Because USPSTF guidelines include women who previously had a fragility fracture, 10 of the 16 studies included women under 65 years who also met USPSTF screening requirements [8, 17, 18, 21, 24–29]. The sample size of the studies varied, from studies as small as 185 participants [18] to as large as 25,783,720 participants [19]. Overall, the aggregate number of patients being analyzed in this review is 27,530,107 patients. Follow-up for patients ranged from 1 to 7 years [10, 16–23, 29], while 6 studies [8, 24–28] did not have any follow-up interval with their patients.

### 3.4. Main Findings

The main findings related to DXA screening are summarized in Table 2. While all studies evaluated DXA screening as an outcome to measure screening disparities, 3 of the 16 chosen studies also measured referrals to receive DXA screening as an outcome [8, 16, 27]. Overall, all but 2 studies demonstrated that disparities existed in DXA screening for racial and ethnic minority populations [18, 22].

### 3.5. DXA Referral Disparities

All 3 studies that evaluated DXA referral rates among racial-ethnic minorities and nonminorities in each population determined that there was a decrease in physician DXA referrals among the racial and ethnic minority populations [8, 16, 27]. One study found that only 29% of eligible African Americans patients were referred for DXA screening, while there was about a 10% increase in DXA referrals in White patients [8]. Miller et al. [16] determined a 23% increase in DXA referrals for African Americans, where 32% of eligible patients were referred, compared to White patients, where 55% were referred for DXA screening. Another study conducted in Israel by Tandeter et al. [27] compared the racial minority group of Ethiopians to non-Ethiopians and determined that only 8% of eligible Ethiopian women received DXA referrals, while 48% of eligible non-Ethiopian women received DXA referrals, a 40% decrease in screening referral rates among Ethiopian patients.

### 3.6. DXA Screening Disparities

All studies chosen for further analysis studied discrepancies in DXA screening rates. Most articles found that there was a difference between racial and ethnic minority and nonminority groups in DXA screening rates. Of the 16 articles, 13 analyzed the disparities between Black and White Americans [8, 10, 16–26, 29]. Of those, 11 found significant disparities in DXA screening, with a range 6% to 61.2% difference in DXA screening rates between Black and White patients, and an average of 23.4% difference in DXA screening rates of Black patients when compared to White patients when Black patients were characterized as the racial-ethnic minority in the population [8, 10, 16–21, 23–26, 29].

The disparities between Hispanic groups and White groups, when Hispanic patients were the racial-ethnic minority in the population, were evaluated in 4 studies [10, 17, 18, 20], of which 3 articles found a decrease in DXA screening [10, 17, 18] and 2 articles [17, 18] identified an average of 5.4% decrease in DXA screening in Hispanic populations when compared to White patients. One study found a 33% decrease in DXA screening rates among Hispanic populations as compared to White patients [10].

One study based in Israel [28] compared DXA screening rates between Arab women (racial-ethnic minority) and Jewish women (racial-ethnic majority). The results indicated that of eligible Arab women, 10.1% received DXA screening compared to Jewish women, of whom 67.6% of eligible women received a DXA screen. As such, the study identified a 57.5% difference in screening rates among racial minority and majority patients.

Two studies did not find any significant difference between the racial minority and majority groups [18, 22]. Becker et al. established an overall lack of DXA screening in all populations, and while the authors determined that factors such as rural residence were associated with decreased care, race was not associated with the decreased quality of care [22]. Another study found higher rates of DXA completion in Black women than White women, with 37% and 33% of eligible women receiving DXA screening, respectively [18]. The authors also determined that only 28% of eligible Hispanic women received DXA screening, further emphasizing this disparity.

Notably, one study [25] that measured both DXA referrals and screening found that while there was a disparity in referral rates, there were similar rates of DXA completion between racial and ethnic minority and nonminorities in those that were referred. However, yet another study showed that not only were African American women less likely to be referred for DXA, but they were also less likely to attend their DXA appointments [8]. One proposed reason by the authors suggested that Black women tended to go to their screening appointments less because they believed that they had less of a chance of developing osteoporosis.

## 4. Discussion

This review aimed to summarize the disparities in osteoporosis screening that exist between racial minority and nonminority groups in hopes of improving clinical care in osteoporosis. Overall, the findings from this review showed that significant disparities do exist, and racial and ethnic minorities receive DXA screening at much lower rates than racial and ethnic majority groups. Ultimately, this may lead to lower rates of osteoporosis diagnosis and subsequent treatment, thus subjecting underrepresented groups to increased morbidity and mortality from osteoporosis and related fractures.

It is important to consider the reasons that these disparities may exist. One study identified that Black patients were referred to DXA screening less than their White counterparts [25]. However, they also determined that there was a similar rate of DXA completion rates in patients that were referred to screening, regardless of race [16]. This discrepancy suggests that the disparity in DXA screening rates, which were still observed within the population, was solely due to the lack of a physician referral to DXA, rather than a lack of patient follow-through. It is possible that physicians may inaccurately believe, consciously or unconsciously, that Black patients are at a lower risk for osteoporosis given that they have a higher BMD compared with Hispanic, White, and adults of Asian descent, ultimately leading to a decrease in physician referral rates. In the article, the authors suggest that lack of referral may be related to physicians erroneously believing that there are differences in bone biology between African American and White patients [16]. Physicians tend to make assumptions based on race without considering other risk factors. For example, one risk for decreased BMD, hypovitaminosis D, is more common in Black patients than in White patients [30].

While not studied specifically in the articles included in this review, another plausible reason for the disparity in osteoporosis screening may involve that initiating education, awareness, and shared decision-making between patient and physician requires time and knowledge about osteoporosis and its risk factors that many physicians are lacking [31]. Often physicians choose to limit preventative care due to time constraints in an already overburdened health care system, with patient's current medical needs taking precedence [31]. Indeed, such is the case for osteoporosis screening. A cognitive error known as the ecological fallacy was explored in Neuner et al. where providers considering fracture prevention may overemphasize the decrease in fracture risk in underrepresented individuals, thereby improperly applying population statistics to single patients [10]. When this occurs, physicians are at a risk of incorrectly assuming a patient of racial and ethnic minority status who may not be at a risk for osteoporosis and fracture and will not spend the time required to appropriately assess fracture prevention. Moreover, many physicians believe that cost and adverse effects of treatment are too great to warrant osteoporosis screening in the population [32], indicating a lack of physician knowledge of the burden of osteoporosis and effectiveness of screening.

Also, physicians may be more likely to engage in these discussions with patients who look like themselves. Most physicians in the United States are White [33] and may have more in depth conversations with their White patients when compared to racial-ethnically underrepresented groups [34]. Multiple studies have found that a racial bias exists among physicians and that specifically Black patients were perceived to be less intelligent and noncompliant, even after controlling for the income and education level [34]. Even among physicians who did not share an explicit bias against underrepresented groups, a distinct implicit bias against people of color has been demonstrated [35]. Patients may also feel more comfortable initiating conversations and advocating for themselves with physicians with whom they identify. While physicians may understand this as a lack of understanding, the long-held mistrust of physicians by people of color may lead patients to ask fewer questions and be less engaged in the patient-physician decision-making process [34]. This may ultimately lead to less shared decision-making and less preventative health services, including DXA screening. Curtis et al. suggested that system-based interventions such as self-referral may prove to be beneficial in increasing the DXA usage rate among underrepresented populations, citing the success that has been shown for self-referral for mammography of at-risk persons [19]. This practice could potentially reduce bias between the physician and the patient.

It is also important to recognize the two studies that did not find any significant differences in osteoporosis screening between different racial groups. Becker et al. determined that ethnic distributions of DXA referral and completion were similar to ethnic distributions within the community [18]. This may be due to the method in which patients were referred, where patients who already had a fragility fracture were identified through a new consultation program and were offered further evaluation. Identifying patients with fragility fracture eliminates bias that may otherwise prevent underrepresented patients from receiving DXA screening. While a method such as this one may succeed in eliminating bias, it does not address primary prevention of a fragility fracture, for which DXA screening is most important. Solimeo et al. studied patients using the US Department of Veterans' Affairs (VA) [22]. The VA, supported through government funding, is uniquely positioned to remove the majority of cost barriers to care, thereby offering additional preventative medicine and more services to patients who may be overlooked in the private sector of healthcare.

### 4.1. Strengths and Limitations

The review is limited in its generalizability as it included studies conducted over a 21-year span. It is important to note that within these 21 years, changes in clinical practice have occurred. Additionally, the political climate regarding race has been changing constantly and could affect the rates of osteoporosis screening in these populations. In particular, the recent Black Lives Matter movement could influence physicians and how they approach their treatment of Black patients. Therefore, the disparities in osteoporosis screening are not static, and the longitudinal timeframe of included studies in this review is a limitation.

While the review was comprehensive in its scope to include all countries and continents, most studies meeting inclusion criteria for this review were conducted in the United States, with two studies conducted in Israel. Thus, we recognize that this systematic review may not be generalizable to other populations. It is essential for healthcare communities in other countries to conduct population-based studies on osteoporosis screening, diagnosis, and treatment to fully gain an understanding of the full disparity worldwide.

This review is strengthened by the high quality of the articles that were included in this study as determined by the quality and risk-of-bias assessment. All studies were observational studies. All studies were either blinded, unblinded with objective measures, or data were collected through secure records, adding to the generalizability of this review. Moreover, sample sizes varied greatly throughout the multiple reports and included many participants, providing an elevated level of power to the review.

The use of two reviewers who were blinded to each other while identifying studies to include, complete quality appraisals, as well as extract and analyze data, also strengthened the review. Furthermore, the authors used a broad range of search terms to ensure that pertinent studies were not missed.

### 4.2. Implications for Practice and Research

The findings of this review introduce significant implications for clinical practice regarding osteoporosis screening. First, physicians and wider organizations must address both implicit and explicit biases that affect the decision-making processes. An effective method of reducing bias involves increasing awareness of bias and developing better habits through practice, feedback, and reflection [35, 36]. This can be accomplished at an institutional level to facilitate the elimination of bias in the healthcare system.

This review only examines osteoporosis screening by DXA. While assumptions are made regarding the sequalae to lack of osteoporosis screening, such as reduced rates of diagnosis and treatment followed by increased fractures and mortality, this has not been explicitly examined. Understanding the consequences of decreased DXA screening in patients of color is critical to supporting policy and public health initiatives in this area. Furthermore, research is needed to prevent and manage osteoporosis in all patient populations equitably. One way to approach this need is for large, population-based, and multiethnic/racial studies to examine the prevention and management of osteoporosis, including DXA screening.

Furthermore, there is a significant lack of research dedicated to assessing the Asian population as it related to disparities in bone health. Specifically, only two of the articles included patients of Asian descent in their studies. When studies did mention the Asian population, there was no discussion surrounding existing disparities. In contrast, while more articles commented on the Hispanic population, there remains a paucity of data among the Hispanic population compared to White and Black populations further highlighting the need for population-based studies that examine screening disparities [37].

## 5. Conclusion

This review provided a summary of articles which revealed a significant disparity among racial and ethnic minorities in osteoporosis screening. This likely leads to further disparity in osteoporosis treatment. Furthermore, research must focus on identifying additional discrepancies in osteoporosis diagnosis and treatment. Moreover, research should identify methods of limiting the known discrepancies of racial and ethnic minorities in osteoporosis screening.

## Figures and Tables

**Table 1:** Inclusion and exclusion criteria.

Inclusion criteria	Exclusion criteria
The population includes men and women 40 years of age and older who meet clinical indications for BMD screening, were referred for BMD screening by a health care provider, and/or experienced a fragility fracture	Papers that were not published between January 2000 and July 2021

The studies are focused on racial and/or ethnic minority status	Papers not written in English

Controls included racial and ethnic nonminorities	Papers that do not study an at-risk population for osteoporosis; studies that focus on adults younger than 50 years old and adolescents

Studies used a referral for BMD screening by dual energy X-ray absorptiometry (DXA); receiving BMD screening (initial or follow-up) by DXA as a measure of outcome	Case reports and reviews will be excluded

Includes observational studies: specifically cross-sectional, cohort, and case control studies and randomized control studies	Studies assessing imaging modalities other than DXA

Studies conducted in a clinical setting, using a medical database or population-based administrative health data. All countries and continents are eligible	Studies that only assess racial or ethnic differences in BMD (T-score) or fracture rates

**Table 2:** Characteristics of included studies.

Author and publication year	Study setting and recruitment	Sample size at pretest	Age (years)	N Female (percentage)	N Male (percentage)	Race or ethnicity minority (percentage)	Race or ethnicity nonminority (percentage)	Measured outcomes	Follow-up duration (years)	Results
Amarnath, 2015	United States, USPSTF	50,995	40–85	50,995 (100)	0	Hispanic 3,278 (6.4) Black 3,021 (5.9) Asian 2,574 (5.0) Other 2,865 (5.6)^∗^ Unknown 13, 826 (27.1)	White 25,431 (49.9)	DXA screening	Average 4.4	Compared to White women, DXA screening was significantly less common among Black women. 15% of eligible Black women received a DXA screen, 19.2% of eligible Hispanic women received a DXA screen, and 25% of eligible White women received a DXA screen

Becker, 2006	United States, Columbia University Medical Center	185	48–99	155 (83)	30 (16)	Hispanic 39 (21) Black 30 (16) Other 2 (1)	White 114 (62)	DXA screening	2	The ethnic distribution of patients receiving DXA scans mirrored the ethnic composition of the entire study population. 37% of eligible Black women received a DEXA scan, 28% of Hispanic women received a DXA scan, and 33% of White women received a DXA scan

Curtis, 2008	United States, Medicare claims data	25,783,720	≥65	15,142,440 (58.7)	10,641,280 (41.3)	Black 1,966,800 (7.6) Other 1,162,400 (4.5)	White 22,654,520 (87.9)	DXA screening	6	Using data from all 7 years observed (1999–2005), the proportion ever tested was 31.3% for White women compared with 15.3% for Black women (*p* < 0.0001). For men, the respective proportions were 4.6% and 1.9% (*p* < 0.0001)

Gillepsie, 2007	United States, USPSTF	1,638,454	≥65	1,638,454 (100)	0	Black, non-Hispanic 174,756 (10.7) Asian, non-Hispanic 45,102 (2.8) Hispanic 112,781 (6.9) Other/not reported 127,272 (7.8)	White, non-Hispanic 1,178,543 (71.9)	DXA screening	2	Even after accounting for socioeconomic status, health status, and healthcare utilization patterns, non-Hispanic Asian and Hispanic women in the 50–64 and 65–79-year age groups had the highest odds of screening, whereas non-Hispanic Black women had the lowest odds across all age groups in our cohort. Among women 50–64 years of age, screening odds for non-Hispanic Blacks were 18% lower compared with non-Hispanic Whites

Gourlay, 2007	United States, North Carolina family medicine research network	400	≥45	400 (100)	0	Black 80 (20)	White 190 (47.5)	DXA screening	—	White women were more likely than Black women to receive DXA. 21.3% of eligible Black women received DXA, while 56.8% of eligible White women received a DXA screen

Hamrick, 2006	Southeastern United States, multiple primary care clinics	739	≥50	546 (100)	0	Black 79 (45.9)	White 452 (51.7)	DXA screening	4	In women 65 years and older with universal screening recommendations, 19.4% (*n* = 46) of the screened women were African American, and 80.6% (*n* = 191) were white. The prevalence of osteoporosis was similar in both populations, 21.5% and 20.1% for African American and White women, respectively

Hamrick, 2012	United States, multiple primary care clinics	1000	≥60	1000 (100)	0	Black 500 (50.0)	White 500 (50.0)	DXA referral, DXA screening	—	Among the initial 1000 women, only 29.8% African American women were referred to DXA compared to 38.4% White women (*p* < 0.05), and 20.8% African American vs. 27.0% White (*p* < 0.05) women completed the test

Lee, 2019	United States, Department of Orthopedic surgery in Virginia Commonwealth University	191	≥50	71 (37.1)	119 (62.3)	Black 65 (34.0) Other 5 (2.6)	White 121 (63.4)	DXA screening	3	Only 13 (6.8%) of all people who underwent hip fracture surgery received DXA screening. Of eligible White patients, 11 (9.1%) received DXA screening while 2 (3.1%) of eligible Black patients received DXA screen

Mikuls, 2005	Unites States, 6 preselected countries in Alabama	251	≥45	251 (100.0)	0	Black 73 (29.0)	White 178 (71.0)	DXA screening	—	Caucasians were substantially more likely (46%) than African Americans patients (19%, *p* < 0.001) to have received a DEXA examination

Miller, 2005	United States, 2 outpatient clinics in Baltimore Maryland	205	≥65	205 (100.0)	0	Black 103 (50.2)	White 102 (49.8)	DXA referral, DXA screening	1	Eighty-nine (43%) of the at-risk women were referred for a DXA scan. Of the 102 White women, 56 (55%) were referred for DXA, while 33 (32%) of the 103 African American women were referred. If referred, African American women had comparable DXA completion rates when compared with White women

Mudano, 2003	United States, Alabama	8,909	≥50	8,909 (100.0)	0	Black 1,960 (22.0)	White 6949 (78.0)	DXA screening	—	Black participants had approximately two-thirds lower odds of receiving BMD testing compared to White participants. 10% of eligible Black women received DXA screening while 25% of eligible, White women received DXA screening

Neuner, 2007	United States, Illinois, Florida, New York	35,681	65–89	35,681 (100.0)	0	Black 1,044 (2.9) Hispanic 598 (1.7)	White 34,039 (95.4)	DXA screening	4	Women of Black race were about half as likely (RR0.52 [0.43, 0.62]) and Hispanic women about 2/3 as likely (RR 0.66 [0.54, 0.80]) as White women to undergo testing before their fracture. They remained less likely (RR 0.66 [0.50, 0.88] and 0.58 [0.39, 0.87], respectively) to undergo testing after fracture

Solimeo, 2019	United States, US Department of Veterans Affairs	7,371	≥65	0	7,371 (100.0)	Black 750 (10.2) “All other” 522 (7.1)	White 5476 (74.8)	DXA screening	7	Age and race were not significantly associated with the receipt of osteoporosis care

Tandeter, 2007	Israel, 3 primary care clinics of Clalit Health Services	347	50–75	347 (100.0)	0	Ethiopian 121 (34.9)	Non-Ethiopian 296 (85.3)	DXA referral, DXA screening	—	The general population received more preventive recommendations and treatment than did Jewish Ethiopian women, including bone density scans. 8% of Ethiopian women received a DXA referral or screen, with 48% of non-Ethiopian women received a DXA referral or screen

Werner, 2005	Israel, a large tertiary medical center	261	≥45	261 (100.0)	0	Arab 79 (30.3)	Jewish 182 (69.7)	DXA screening	—	Compared with Jewish participants, a lower percentage of Arab women had bone density examinations. 10.1% of Arab women received DXA screen, while 67.6% of Jewish women received DXA screen

Yoo, 2012	United States, US Urban Area Health System	1,398	≥65	1398 (100.0)	0	Black 825 (59.0)	White 573 (41.0)	DXA screening	3	Significantly fewer Black than White female Medicare beneficiaries received the DXA screening. 26% of Black women received screening, while 33% of White women received screening

^*∗*^includes native American, Alaska native, native Hawaiian, other pacific Islander, North African, Iran, other Asian/Mideastern, or others.

**Table 3:** Quality appraisal of selected articles. Quality appraisal was completed using the Newcastle-Ottawa Scale for case control and cohort studies and the modified Newcastle Ottawa Scale for cross-sectional studies.

Study author, year	Study type	Quality appraisal score
Amarnath, 2015	Cohort	7
Becker, 2006	Cohort	7
Curtis, 2008	Cohort	8
Gillepsie, 2007	Cohort	8
Gourlay, 2007	Cross-sectional	8
Hamrick, 2012	Cohort	9
Hamrick, 2006	Case control	9
Lee, 2019	Cohort	7
Mikuls, 2005	Cross-sectional	8
Miller, 2005	Cohort	8
Mudano, 2003	Cross-sectional	9
Neuner, 2007	Cohort	8
Solimeo, 2019	Cohort	8
Tandeter, 2007	Cross-sectional	8
Werner, 2005	Cross-sectional	8
Yoo, 2012	Cohort	8

## Data Availability

All data generated or analyzed during this study are included within the article.
